# Dietary Patterns, Food Intake and Health: New Evidence from Epidemiological and Genetic Studies

**DOI:** 10.3390/nu16070919

**Published:** 2024-03-22

**Authors:** Qian Yang, Yangbo Sun

**Affiliations:** 1 MRC Integrative Epidemiology Unit, University of Bristol, Bristol BS8 2BN, UK; qian.yang@bristol.ac.uk; 2 Population Health Sciences, Bristol Medical School, University of Bristol, Bristol BS8 1UD, UK; 3 Department of Preventive Medicine, The University of Tennessee Health Science Center, Memphis, TN 38163, USA

Our special issue gathered 11 articles in the field of nutritional epidemiology, some of which applied newly developed statistical methods to make causal inference. Below is an overview of them.

## 1. Dietary Patterns

Dietary patterns play an essential role in shaping overall health. Mateo-Orcajada and colleagues investigated levels of adherence to Mediterranean diet—one of the healthiest dietary patterns [1], while both Hu et al. and Marinoni et al. applied principal component analyses to identify specific dietary patterns among the participants [2,3]. Specifically, characteristics of physical activity and anthropometric measurements among 791 Spanish adolescents were described, categorized by levels of adherence to Mediterranean diet and body mass index (BMI) [1]. Hu et al. found that women’s adherence to wheaten food-coarse cereals pattern in 21–24 gestational weeks was associated with a lower risk of gestational hypertension in one subgroup [2], although an association did not show a clear dose-response relationship among all 1082 participants, which requires further validations and subsequent investigations of potential mechanisms. Results from Marinoni’s cross-sectional study of dietary patterns on cognitive performance in 379 Italian children showed that a concurrent diet based on fish consumption may be associated with better verbal abilities and perceptual reasoning, diets based on meat and potatoes were associated with worse verbal abilities, and diets based on dairy products were associated with reduced processing speed [3].

Another three articles paid attention to specific food items, including coffee, added sugars, and fish oil. Lee JY and colleagues systematically reviewed existing evidence on the association of coffee intake with irritable bowel syndrome, and meta-analysed eight observational studies with fixed effect models [4]. Lee SH et al. described characteristics of added sugars consumers in National Health and Nutrition Examination Survey (NHANES) 2015–2018—a large nationally representative nutrition and health survey among US adults, and showed that the major source of added sugars was sweetened beverages [5]. Habitual consumption of sugar sweetened beverages has been shown to be associated with increased all-cause mortality and risks of cardiometabolic diseases [6,7]. Effectiveness of public health policies targeting on reducing sugar sweetened beverages consumption (e.g., sugar tax) should be evaluated. Yildiz and Medina used both animal and human models to investigate the effect of fish oil supplementation on cerebral metabolic rate of glucose in multiple areas of ageing brains, and emphasised their application of thermodynamic analysis [8].

## 2. Human Genetics

In 1986, Martjin B Katan first proposed using genetic variants controlling apolipoprotein E synthesis instead of measured plasma cholesterol to estimate causal association of cholesterol on cancer [9]. This idea was further developed as Mendelian randomization (MR)—an instrumental variable analysis using genetic information as the instrument [10]. In this special issue, three studies have used human genetic data to explore the association between dietary factors and health. Sugimoto et al., explored associations of genetic variant rs671 located in ALDH2 gene with a range of variables among 1612 Japanese men and women [11]. ALDH2 variant is non-rare in East Asian participants, and associated with approximately 10–15 g alcohol intake per effect allele per day [12]. Among European participants, it is a rare variant, and thus researchers tended to use rs1229984 located in ADH1B gene as an instrument for MR analyses [13], as both genes encode for enzymes in alcohol metabolism. Notably, these genes have a gene-by-environment phenomenon, i.e., they can only proxy alcohol intake among ever drinkers but would not work among never drinkers [14]. Kafyra et al. examined the interactions between vascular endothelial growth factor A (VEGFA) gene and dietary patterns, and explored their associations with cardiometabolic factors among 766 participants in Greek TEENAGE study [15]. Gene-by-environment MR has been further developed to use offspring genotypes as proxies for unmeasured maternal genotypes, in order to explore offspring intrauterine exposures on their later life outcomes [16].

Recent MR studies set out to obtain effect size estimation for associations of dietary-related exposures (rather than their genetic instruments) with health outcomes. Feng et al. identified 43 genetic variants genome-wide significantly associated with vegetable intake from previous genome-wide association studies, and found little evidence for associations of vegetable intake with cardiovascular diseases by using those variants as instruments [17]. In MR studies of food items, selection of instruments reflecting biological rationale is fundamentally important, as what has been done in Feng’s study. For example, the lactose persistence gene has been reasonably and widely used to instrument milk consumption [18]. However, MR studies without further validating biological pathways from their instruments to the exposure of interest (e.g., noodle intake) should be interpreted with caution. Adherence to the STROBE-MR criteria with publication of its author-completed checklist [19], would be helpful to improve the quality of MR studies in nutritional epidemiology.

## 3. Obesity

This special issue published two studies focusing on risk factors for obesity in childhood and adolescence. Su et al. found time of computer use was inversely associated with overweight and obesity among 513 children but little evidence for 1286 adolescents in the China Health and Nutrition Survey (CHNS) 2004–2011, after adjusting for potential confounders, e.g., energy intake [20]. Zhang et al. conducted a longitudinal analysis of maternal pre-pregnancy BMI on offspring obesity in Avon Longitudinal Study of Parents and Children (ALSPAC), and showed that age of weaning may not be a key mediator [21]. Maternal pre-/early pregnancy BMI is known to have short-term adverse effects on a range of perinatal outcomes, but its long-term effects on offspring still require investigations [22,23].

In summary, articles in this special issue explored associations of dietary patterns/items on health, and identified potential risk factors for obesity, taking advantages of publicly available data resources (e.g., NHANES and CHNS) and large cohorts/biobanks (e.g., UKB and ALSPAC). To further make causal inference for these observed associations, we recommend triangulating evidence from multiple study designs that rely on different assumptions and thus different sources of bias [24].
